# From gastroenteritis to myocarditis: a case series of Campylobacter-mediated cardiac involvement

**DOI:** 10.1093/ehjcr/ytaf003

**Published:** 2025-01-09

**Authors:** Ricardo Craveiro Costa, Maria Ribeiro Estevens, Marta Correia, Cláudia Cristóvão, Duarte Saraiva Martins, Hugo Castro Faria

**Affiliations:** Pediatrics Department, Unidade Local de Saúde de Coimbra, Avenida Dr. Afonso Romão 3000-602 Coimbra, Portugal; Child and Adolescent Center, Hospital CUF Descobertas, R. Mário Botas S/N, 1998-018 Lisboa, Portugal; Department of Pediatric Cardiology, Hospital de Santa Cruz, Centro Hospitalar Lisboa Ocidental, Rua da Junqueira 126, 1349-019 Lisboa, Portugal; Intermediate Care Unit, Child and Adolescent Center, Hospital CUF Descobertas, R. Mário Botas S/N, 1998-018 Lisboa, Portugal; Child and Adolescent Center, Hospital CUF Descobertas, R. Mário Botas S/N, 1998-018 Lisboa, Portugal; Department of Pediatric Cardiology, Hospital de Santa Cruz, Centro Hospitalar Lisboa Ocidental, Rua da Junqueira 126, 1349-019 Lisboa, Portugal; Child and Adolescent Center, Hospital CUF Descobertas, R. Mário Botas S/N, 1998-018 Lisboa, Portugal; Intermediate Care Unit, Child and Adolescent Center, Hospital CUF Descobertas, R. Mário Botas S/N, 1998-018 Lisboa, Portugal

**Keywords:** *Campylobacter jejuni*, Myocarditis, Echocardiography, Gastroenteritis, Cardiac magnetic resonance imaging, Case series

## Abstract

**Background:**

While viruses remain the leading cause of infectious myocarditis, improved diagnostic methods have highlighted the role of bacteria as a possible cause. We report two cases of myocarditis as a complication of *Campylobacter jejuni* infection.

**Case summaries:**

Patient A, a 17-year-old Caucasian male with a history of asthma, presented to the emergency department (ED) after experiencing fever and nausea for four days, followed by 1 day of diarrhoea and chest discomfort. Laboratory evaluation revealed elevated troponin levels. Transthoracic echocardiography showed left ventricular enlargement and apical dyskinesia. *C. jejuni* was identified in stool cultures. Cardiac magnetic resonance imaging confirmed the diagnosis of myocarditis. The patient was treated with furosemide and enalapril, with improvement of symptoms. Patient B, a previously healthy 14-year-old Caucasian male, presented to the ED with retrosternal chest pain lasting 2 h. He also reported a 3-day history of fever, nausea, and diarrhoea. Electrocardiography showed widespread PR-segment depression and concave ST-segment elevation. Laboratory testing revealed elevated Troponin I levels, and *C. jejuni* was identified in stool cultures. Cardiac magnetic resonance imaging findings were consistent with acute myocarditis. The patient was treated with ibuprofen and azithromycin, leading to resolution of symptoms. Eight months later, he returned with recurrent chest pain and dry cough. Cardiac magnetic resonance imaging at this time showed T1 and T2 criteria consistent with recurrent myocarditis.

**Discussion:**

Although rare, clinicians should be aware of the potential cardiac involvement in patients with Campylobacter gastroenteritis, paying special attention to myocarditis symptoms like chest pain or shortness of breath, especially in areas with elevated Campylobacter infection rates.

**Learning points**

- It is essential to recognize the signs and symptoms of myocarditis, which can be subtle and mimic other cardiac conditions. Campylobacter infections should be considered as a possible cause of myocarditis, especially in areas with high rates of Campylobacter infections.
- Cardiac magnetic resonance imaging plays a pivotal role in the evaluation of myocarditis by enabling visualization of inflammation within the heart. This imaging modality is instrumental in assessing the extent and severity of myocardial involvement, including the identification of areas of necrosis and fibrosis. Such findings are not only essential for diagnostic purposes but also serve as important prognostic indicators, guiding treatment strategies and predicting patient outcomes.
- The management of myocarditis encompasses a spectrum of interventions, ranging from supportive care to the consideration of immunosuppressive therapies in select cases. Given the unpredictable nature of the recovery process in myocarditis, characterized by potential relapses in some individuals, long-term monitoring and comprehensive care are imperative. This underscores the necessity for healthcare providers, particularly cardiologists, to adopt a multidisciplinary approach and ensure ongoing surveillance to optimize patient outcomes and quality of life.

## Introduction

While viruses remain the leading cause of infectious myocarditis, improved diagnostic methods have highlighted the role of bacteria such as Salmonella, Shigella, and, more rarely, Campylobacter.^1^ Campylobacter is a gram-negative bacterium commonly associated with gastrointestinal (GI) infections, but its involvement in myocarditis, although infrequent, has been increasingly recognized.^1,2^ The exact mechanisms through which Campylobacter induces myocarditis remain unclear, but it is believed that the infection may lead to an inflammatory response that affects the heart, either through direct invasion or via immune-mediated pathways.^1,2^ This highlights the importance of considering Campylobacter as a potential cause of myocarditis, particularly in patients with GI symptoms. We present two cases of Campylobacter-associated myocarditis (CAM) to illustrate the diverse clinical presentation and diagnostic challenges associated with this condition.

## Summary figure

**Figure:**
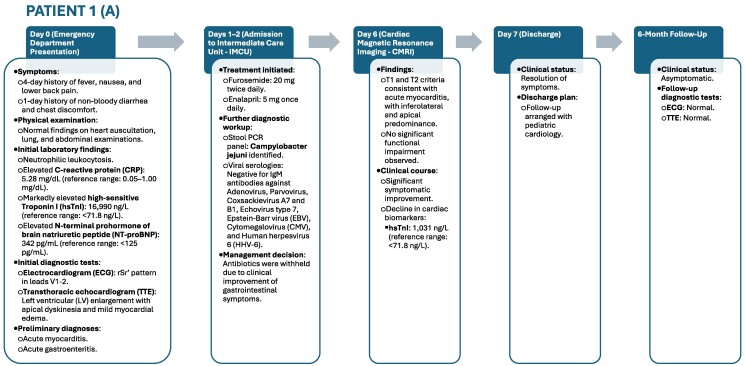

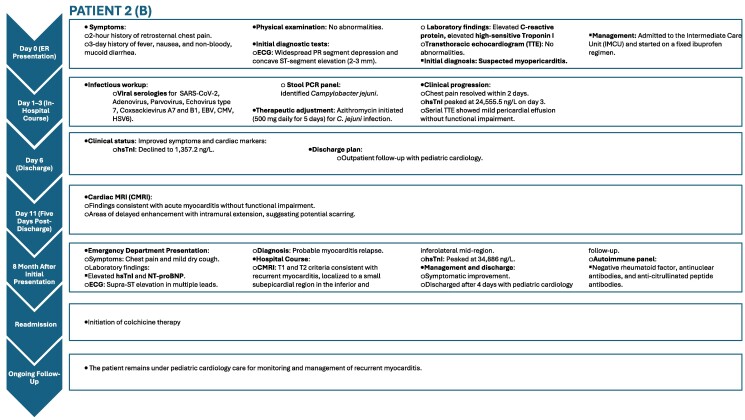


## Case presentations

### Patient 1

A 17-year-old Caucasian male (A), with a previous history of asthma, presented to the emergency department (ED) with a 4-day history of fever, nausea, and lower back pain, and 1-day history of non-bloody diarrhoea and chest discomfort. Vital signs were stable, with normal circulatory and respiratory parameters. Heart auscultation was normal. Lung and abdominal examinations were also normal. Laboratory testing showed neutrophilic leukocytosis, increased levels of C-reactive protein (5.28 mg/dL, reference range: 0.05–1.00 mg/dL), high-sensitive Troponin I (hsTnI) (16 990 ng/L, normal value: <71.8 ng/L), and N-terminal prohormone of brain natriuretic peptide (NT-proBNP) (342 pg/mL, normal value: <125 pg/mL). Polymerase chain reaction (PCR) for severe acute respiratory syndrome-coronavirus 2 (SARS-CoV-2) on nasopharyngeal swab was negative. The electrocardiogram (ECG) showed rSr’ pattern on leads V1–2 and the transthoracic echocardiogram (TTE) revealed left ventricular enlargement with apical dyskinesia and mild myocardial oedema. The patient was diagnosed with acute myocarditis and acute gastroenteritis and was admitted for inpatient treatment at our intermediate care unit (IMCU). He was treated with furosemide (20 mg 2id) and enalapril (5 mg id). In the following days, he was diagnosed with *Campylobacter jejuni* gastroenteritis after a positive stool PCR panel. Other viral serologies were performed and revealed negative IgM for several viruses, including Adenovirus, Parvovirus, Coxsackie virus A7 and B1, Echovirus Type 7, Epstein–Barr virus (EBV), Cytomegalovirus (CMV), and Herpes simplex 6 (HSV6). In light of the provided patient clinical history, no toxicological screening was performed.

**Figure 1:**
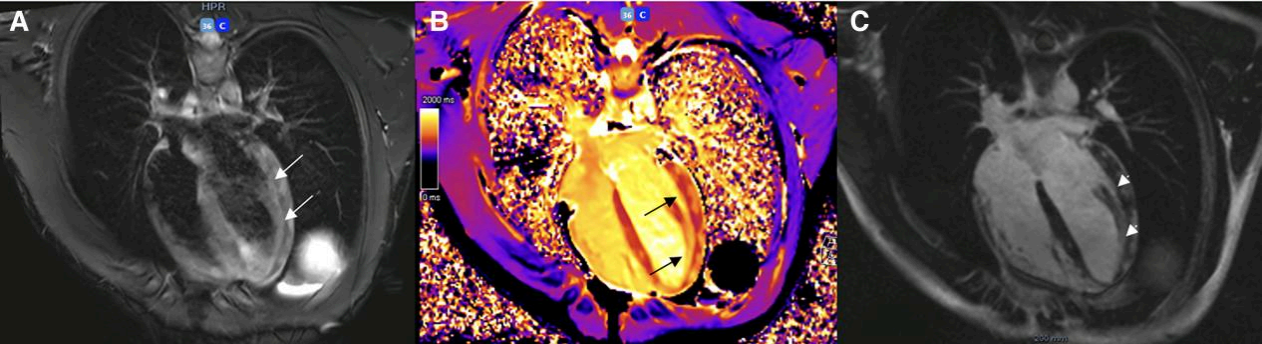
Cardiac magnetic resonance imaging images (four-chamber view) of Patient 1, consistent with acute myocarditis (Lake Louise Criteria fulfilled). (*A*) Short tau inversion recovery image shows several areas of hyperintensity on the lateral wall of left ventricle (white arrows), indicating myocardial oedema. (*B*) T1 mapping image depicts multiple regions of increased myocardial T1 relaxation times (black arrows), reflecting myocardial injury. (*C*) Late gadolinium enhancement sequence shows extensive non-ischaemic late gadolinium enhancement on the same locations (arrowheads), indicating myocardial fibrosis.

On the sixth day of admission, cardiac magnetic resonance imaging (CMRI) revealed T1 and T2 criteria supporting the diagnosis of acute myocarditis, with inferolateral and apical predominance and no functional repercussions (*Figure 1*). After symptomatic improvement and a decline of cardiac markers (hsTnI of 1031 ng/L, normal value: <71.8 ng/L), the patient was discharged with a follow-up by paediatric cardiology. Antibiotic therapy was deemed unnecessary as he began to show signs of clinical improvement with supportive care alone, consistent with the typically self-limiting course of Campylobacter infections in otherwise healthy individuals. The patient had no symptoms throughout the follow-up, and at the 6-month mark, the ECG remained normal, and on the TTE, the dyskinesis has resolved, and normal function had been restored.

### Patient 2

A 14-year-old Caucasian male (B), with no relevant prior medical history, presented to the ED with a 2-h history of retrosternal pain. He had nausea, non-bloody/mucoid diarrhoea, and fever for the last 3 days. Physical examination revealed no abnormalities, but an ECG was typical of pericarditis with widespread PR--segment depression and concaved ST-segment elevation (2–3 mm). Laboratory findings revealed elevated C-reactive protein (7 mg/dL, normal values: 0.05–1.00 mg/dL) and hsTnI levels (2508.8 ng/L, normal value: <71.8 ng/L), while the hemogram, leucogram, and NT-proBNP were normal. Upon suspicion of myopericarditis, a TTE was conducted, showing no abnormalities. The patient was admitted to the IMCU and started ibuprofen in a fixed regimen. In the course of infectious investigations, SARS-CoV-2 PCR on a nasopharyngeal swab yielded negative results, and viral serologies (Adenovirus, Parvovirus, Echovirus type 7, Coxsackie virus A7 and B1, EBV, CMV, and HSV6) were all negative for IgM. In light of the patient's clinical history, no toxicological screening was performed, and subsequently, a PCR panel on the stools identified *C. jejuni*, prompting the initiation of azithromycin at a dosage of 500 mg daily for a duration of 5 days.

**Figure 2:**
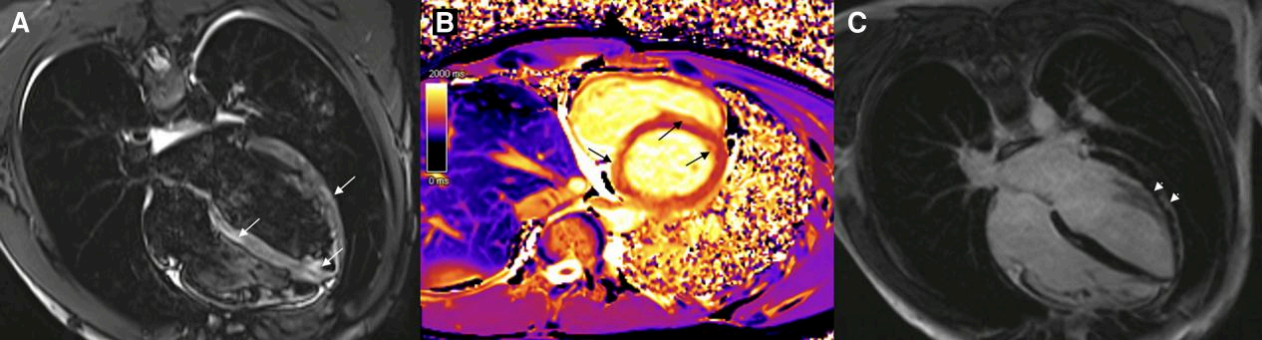
Cardiac magnetic resonance imaging images illustrating acute myocarditis in Patient 2. (*A*) Short tau inversion recovery image in a four-chamber view shows focal areas of hyperintensity on the septal and lateral walls of the left ventricle (white arrows), consistent with myocardial oedema. (*B*) T1 mapping image in a short-axis view highlights elevated T1 relaxation times (black arrows) in the myocardial tissue, indicating widespread myocardial injury. (*C*) Late gadolinium enhancement sequence in a four-chamber view reveals non-ischaemic late gadolinium enhancement at the septal and lateral segments (arrowheads), suggestive of myocardial fibrosis.

The patient remained stable throughout the hospitalization with no alterations in his heart rhythm. His chest pain subsided over the first 2 days, and his hsTnI level peaked at 24 555.5 ng/L on the third day. Serial echocardiograms showed only a mild pericardial effusion, without functional impairment. The patient's symptoms and cardiac markers progressively improved throughout his hospital stay, with the last hsTnI value measuring 1357.2 ng/L. The patient was discharged on the sixth day with instructions to continue follow-up appointments with paediatric cardiology. Five days after discharge, the patient had a CMRI (*Figure 2*) that showed signs of acute myopericarditis, with no impairment in heart function. It also revealed areas of delayed enhancement extending within the heart wall (intramural), suggesting that scarring might persist during future follow-up.

Throughout a 6-month follow-up, the patient remained asymptomatic, with normal serial ECGs and no other complications, except for contracting coronavirus disease 2019 (COVID-19) during this period.

After 8 months (∼2 months post-COVID-19), Patient B returned to the emergency department complaining of chest pain and a mild dry cough. Laboratory testing showed elevated troponin and NT-proBNP levels and ECG with a supra-ST-elevation in several leads. Suspecting a probable relapse of myocarditis, the patient was admitted to the IMCU for further evaluation, and a repeat CMRI was scheduled, which showed evidence of T1 and T2 criteria for recurrent myocarditis involving a small subepicardial region in the inferior and inferolateral mid-region. During this hospitalization, the patient's hsTnI levels peaked at 34 886 ng/L. With consistent symptom improvement, discharge took place 4 days after admission, with a scheduled appointment with paediatric cardiology. Before discharge, an autoimmune study was performed, including rheumatoid factor, antinuclear antibodies titres, and anti-peptide citrullinated antibodies, which were negative.

The patient was readmitted several days after discharge due to persistent chest pain, and the medical team decided to initiate colchicine therapy. The patient remains under the care of paediatric cardiology for ongoing monitoring and management.

## Discussion

Myocarditis is often associated with viral infections but can also result from bacterial infections or other causes.^1,3–5^ The pathophysiology of myocarditis is only partially understood, but several theories exist, including direct bacterial invasion of myocardial tissue, immune-mediated responses, and molecular mimicry.^1,6^ Direct bacterial invasion induces local inflammation and necrosis, leading to myocardial dysfunction and potentially irreversible damage; immune-mediated reactions involve the release of cytokines and antibodies, which can contribute to myocardial injury; and molecular mimicry occurs when the bacterium's structural components resemble host self-antigens, leading to an immune response that attacks the myocardial tissue, resulting in myocarditis.^5–8^

Campylobacter-associated myocarditis is a rare condition documented in only a few case reports.^1,3^ It can manifest as a range of symptoms, including chest pain, palpitations, dyspnoea, and heart failure.^3–5,7,8^ The diagnosis of CAM can be challenging due to the diversity of its clinical presentation and the limitations of current diagnostic methods, which can delay appropriate management, leading to adverse outcomes.^3–5,7,8^

The presented cases describe previously healthy male adolescents who developed myocarditis following Campylobacter infection. According to the available literature, this pathology is more commonly observed in male adolescents.^1^ The patients presented with GI symptoms, fever, and chest pain and were diagnosed with cardiac involvement based on elevated cardiac biomarkers, abnormal ECG findings, and cardiac imaging results. In cardiology, the Dallas criteria are still used to diagnose myocarditis, but their effectiveness is limited by sampling errors, varying interpretations, and poor alignment with markers of viral infection and immune activity.^4,8–10^ As a result, there has been a shift from relying on histopathology to using imaging techniques, which provide more accurate guidance for treatment and predicting outcomes.^4,8–10^ According to these criteria, a definitive diagnosis of myocarditis requires an endomyocardial biopsy (EMB).^4,8,9^ However, a joint statement from the American Heart Association, American College of Cardiology Foundation, and European Society of Cardiology recommends using EMB only when the benefit of performing the biopsy outweighs the risk of complications, often not the case with CAM due to its relatively good prognosis.^8,9,11^ Hence, based on the available literature, it is strongly recommended that EMB not be considered an appropriate diagnostic tool for this specific condition.^4,8,9,11^

The diagnosis of myocarditis using CMRI is crucial for accurate assessment and standardization.^9,11^ To achieve this, consensus criteria known as the Lake Louise Criteria have been established.^11^ The criteria were initially defined in 2009 and were recently updated in 2018 to incorporate advancements in imaging technology and new evidence.^11^ The criteria involve the assessment of myocardial oedema, hyperaemia, and capillary leak, as well as myocyte necrosis and fibrosis. The presence of any two out of the three main criteria indicates a positive imaging diagnosis of myocarditis.^11^ The inclusion of parametric mapping in the updated criteria reflects the advancements in CMRI technology and its ability to detect myocardial inflammation.

Treatment of myocarditis is usually mainly supportive,^1^ and there is no specific antiviral or anti-inflammatory treatment for most cases. In both cases, the patients were managed with medications to control their symptoms and improve cardiac function, including analgesics, diuretics, and angiotensin-converting enzyme inhibitors. Although Patient A was diagnosed with myocarditis, beta-blocker treatment was avoided due to a previous history of bronchospasm episodes. In the case of Patient B, the myocardial involvement was considered a complication of pericarditis, and therefore, he was evaluated and treated accordingly. In cases of CAM associated with GI symptoms or in those who develop significant heart failure, antimicrobial therapy appears potentially helpful to eliminate the underlying infection and prevent disease progression.^6,8,9,12^ Currently, fluoroquinolones and macrolides are the first-line antibiotics used to treat Campylobacter infections, although the emergence of antibiotic resistance in Campylobacter species is a growing concern.^1,8^ The patient in Case 1 was not treated with antibiotics, while Patient 2, who presented almost 2 years later, was started on azithromycin; this difference shows the lack of concrete guidelines on how to treat complicated Campylobacter infections and the diversity of approaches from different clinicians.^8^

The prognosis of myocarditis depends on the severity of the initial presentation and the degree of myocardial injury.^6,8,9^ In both cases, the patients had elevated cardiac biomarkers and abnormal echocardiogram findings, which suggested myocardial injury, confirmed by CMRI. However, only one showed relapsing features on follow-up, requiring colchicine to prevent further cardiac damage. It is important to note that relapses are not unexpected in pericarditis. Therefore, it appears essential to closely monitor patients with myocarditis, even if they initially present with mild symptoms, as the course of the disease can be unpredictable and may require long-term management.

## Conclusion

Myocarditis is an increasingly recognized condition among adolescents and CAM, a rare, yet severe, Campylobacter infection complication requires immediate recognition and appropriate management. Clinicians should be aware of the potential cardiac involvement in patients with Campylobacter gastroenteritis, paying special attention to myocarditis symptoms like chest pain or shortness of breath, especially in areas with elevated Campylobacter infection rates. Additionally, GI symptoms like abdominal pain, vomiting, and diarrhoea can function as early indicators of reduced cardiac output, and, when encountering myocarditis with concomitant GI symptoms and the absence of other signs suggesting reduced cardiac output, it is advisable to cautiously consider the potential diagnosis of CAM. Moreover, careful follow-up is crucial due to potential, although rare, myocarditis relapses.

## Lead author biography


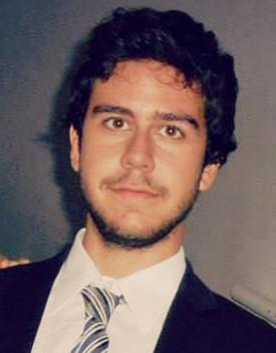


Ricardo Craveiro Costa is a dedicated senior paediatrics resident from Portugal. Currently working at Hospital Pediátrico, Unidade Local de Saúde de Coimbra in Coimbra, and Hospital CUF Descobertas in Lisbon. His interests span general paediatrics, infectious diseases, immune dysfunction, and paediatric rheumatology.

##  

**Consent:** The authors confirm that written consent for submission and publication of this case report including images and associated text has been received from the patient in line with the Committee on Publication Ethics (COPE) guidelines.

**Funding:** None declared.

## Data Availability

The data underlying this article will be shared on reasonable request to the corresponding author.
